# Risk Factors and Outcomes Stratified by Severity of Acute Kidney Injury in Malaria

**DOI:** 10.1371/journal.pone.0090419

**Published:** 2014-03-13

**Authors:** Kavitha Saravu, Kumar Rishikesh, Chirag R. Parikh

**Affiliations:** 1 Department of Medicine, Kasturba Medical College, Manipal University, Manipal, Karnataka, India; 2 Section of Nephrology, Yale University School of Medicine, VA CT Healthcare System, and the Program of Applied Translational Research, New Haven, Connecticut, United States of America; Centro de Pesquisa Rene Rachou/Fundação Oswaldo Cruz (Fiocruz-Minas), Brazil

## Abstract

Severe acute kidney injury (AKI) is known to have prognostic value for in-hospital outcomes in malaria. However, little is known about the association of AKI of lesser severity with malarial risk factors and outcomes – and such a gap is becoming increasingly relevant with the upsurge in the incidence of AKI due to *Plasmodium falciparum* malaria and *Plasmodium vivax* malaria over the last decade. We aimed to identify risk factors of AKI in malaria and assessed in-hospital outcomes stratified by severity of AKI. We performed an observational study of 1,191 hospitalized malaria patients enrolled between 2007 and 2011 in a tertiary care academic center in India. Patients were categorized based on peak serum creatinine into one of three groups: no AKI (<1.6 mg/dL), mild AKI (1.6–3.0 mg/dL), and severe AKI (>3 mg/dL). *Plasmodium vivax* was the predominant species (61.41%), followed by *Plasmodium falciparum* (36.41%) and mixed infections with both the species (2.18%). Mild and severe AKI were detected in 12% and 5.6% of patients, respectively. Mild AKI due to *Plasmodium vivax* (49%) and *Plasmodium falciparum* (48.5%) was distributed relatively equally within the sample population; however, cases of severe AKI due to *Plasmodium falciparum* (80%) and *Plasmodium vivax* (13%) was significantly different (P<0.001). On history and physical examination, risk factors for AKI were age, absence of fever, higher heart rate, lower diastolic blood pressure, icterus, and hepatomegaly. The only laboratory parameter associated with risk of AKI on multivariate analysis was direct bilirubin. Patients with mild and severe AKI had greater organ complications, supportive requirements, longer duration of hospital stay and in-hospital mortality in a dose-dependent relationship, than patients with no AKI. Mild AKI is associated with significant (P<0.05) morbidity compared to no AKI, and future studies should assess strategies for early diagnosis of AKI and prevent AKI progression.

## Introduction

There are more than 3.3 billion people living in countries with ongoing malaria transmission at risk of infection [1]. According to the WHO 2012 report, an estimated 219 million cases of malaria and 600,000 deaths occurred in 2010 [2] due to complications. Acute kidney injury (AKI) is a fairly common and serious complication seen in acute *falciparum* malaria in adults and older children. Depending on the definitions used for AKI, intensity of malaria transmission, age affected, infecting species, and the cohort studied, incidence of AKI in malaria varies from 0.4% to 60% [3], [4]. Over the last decade, there has been an upsurge in the incidence of AKI due to *Plasmodium falciparum(P. falciparum)* malaria [5], [6], [7] and reports of AKI due to *Plasmodium vivax (P. vivax)* malaria [8], [9]. Moreover, in certain parts of the world, AKI associated with malaria is the leading cause of hospitalization due to AKI [6].

The mortality of patients with AKI varies depending on the health care access and availability and has ranged between 10% and 75% in prior studies [4], [10]. Although the mortality associated with severe AKI in malaria is well established, the prognostic importance of less severe forms of AKI is not known. In view of the high mortality rates associated with AKI, it is critical to identify the predictors of AKI in malaria and diagnose renal involvement early to avoid the progression to severe AKI. There has been little work investigating associated factors of severe AKI in malaria. Furthermore, of the published literature, the studies have either been small [4], dated back at least a decade [4], [6], lacking specificity of AKI severity and independent variables [4], [6], or devoid of multivariate analysis [5], [11].

The objective of our study was to conduct a rigorous analysis of associated factors of AKI and elucidate the contribution of the severity of AKI to other organ complications and in-hospital death in malaria.

## Methods

### Study Design and Patients

We performed a retrospective cohort study by extracting information in a data abstraction tool from adult malaria patients, who were hospitalized at a tertiary care teaching hospital in India from January 2007 to December 2011. Adults (≥18 years) of either gender and microscopically proven to have asexual forms of *P. vivax, P. falciparum,* or both with or without gametocytes were included. Only cases with body mass index (BMI) >16 Kg/M^2^ were included because patients with lower BMIs would have low lean body mass and baseline serum creatinine. Cases with other non-malarial fever etiology, coexisting human immunodeficiency virus infection, and pre-existing chronic kidney diseases by history were excluded from this study. All laboratory tests for in-patients of our tertiary care hospital are done in associated laboratories within hospital. Patients were managed by the treating physicians as per their clinical judgement and national guidelines for the treatment of malaria. All patients with AKI were managed with intravenous fluids, diuretics, systemic alkalinisation and hemodialysis as per the decision of attending clinicians in consultation with nephrologists.

### Ethics Statement

Approval for retrospective extraction of all patients’ data from their clinical records and further analysis was granted by our parent institutional (Kasturba Medical College, Manipal University, Manipal, India) ethics committee prior to commencement of this study. For our retrospective study, individual patients’ informed consent was not sought by our parent institutional ethics committee, and all patients’ records were kept anonymous. We would share our deidentified study data upon request after necessary institutional approvals have been obtained.

### Variables

#### Independent variables

Malarial complications were defined based on the guidelines for management of severe malaria by the WHO in 2012 [1]. Cerebral malaria was defined as impaired consciousness, coma, or multiple generalised convulsions within 24 hours. Severe anemia was defined as Hb<7 g/dL; jaundice as total bilirubin>3 mg/dL; hyperparasitemia as parasite index>5% (parasite index was expressed as percentage of parasitized erythrocytes on Leishman’s stained peripheral blood smear); pulmonary edema/Acute Respiratory Distress Syndrome (ARDS) as bilateral spreading shadows on chest x-rays.

#### Dependent variables

Our primary outcome was AKI, defined as serum creatinine ≥1.6 mg/dL. We divided the cohort into three categories based on the peak serum creatinine value during hospitalization; serum creatinine <1.6 (No AKI), 1.6–3.0 (Mild AKI) and >3 mg/dL (Severe AKI). The severe AKI category corresponded to the WHO renal failure definition of creatinine >3 mg/dL (>265 µmol/L) any time during hospitalization. Any patient who required in-patient renal replacement therapy was classified into the severe AKI category. The secondary outcome was in-hospital mortality.

#### Statistical analysis

Categorical data were summarized as frequency and proportions by AKI categories; proportions were compared by the Chi-square test or Fisher’s exact test as appropriate. Continuous variables were tested for normality using the Kolmogorov-Smirnov test. Normally distributed continuous variables were reported as means with standard deviations, and comparisons of groups were performed by analysis of variance (ANOVA). Non-normally distributed data were summarized as median s with interquartile range (IQR) and compared by the Kruskal-Wallis test. Univariate analysis was performed with baseline characteristics, clinical variables as independent variables, and AKI as the dependent variable to determine the possible associated factors for AKI among malaria patients. Variables achieving a value ≤0.1 in the univariate logistic regression analysis were selected and checked for multicollinearity. If multicollinearity was present, only one of the factors would be selected and included in the multivariate logistic regression analysis. All tests of significance were two-sided, with a p-value of <0.05 indicating statistical significance. The data were analysed using the Statistical Package for the Social Sciences version 16.0 (SPSS, Chicago, IL, USA).

## Results

A total of 1,191 patients with malaria were hospitalized between January 2007 and December 2011. Medical records of 109 (9.15%) patients could not be obtained, and 232 (19.48%) cases were excluded due to pre-specified criteria as follows: age <18years –96 patients, microscopically negative but rapid diagnostic test (RDT) positive malaria cases –87 patients, cases with concomitant febrile illnesses (dengue, hepatitis ‘B’, hepatitis ‘C’, HIV, leptospirosis, filariasis, acinetobacter & *Staphylococcus aureus* sepsis, typhoid fever, paratyphoid fever, tuberculosis, UTI, neurocysticercosis and chronic obstructive pulmonary disease and lower respiratory distress syndrome due to *Pseudomonas aeruginosa* infection –39 patients, cases with BMI<16 kg/M^2^–6 patients, and 4 patients due to chronic kidney diseases. Of the 850 who fulfilled the eligibility criteria, serum creatinine values were not available in 26 patients, and the remaining 824 patients were analyzed. *P. vivax* was the predominant species causing malaria, responsible for the cases of 61.41% of the cohort, whereas *P. falciparum* and mixed infection with *P. vivax* and *P. falciparum* accounted for 36.41% and 2.18%, respectively.

### Demographic, Clinical, and Laboratory Characteristics across AKI Categories for All Malaria Cases

Among the study cohort, 677 (82.16%) patients had no AKI, 101 (12.26%) patients had mild AKI, and 46 (5.58%) patients had severe AKI. The demographic and clinical characteristics of the groups are given in Table 1. Males contributed to >80% of cases in all the categories. Patients with AKI were older than those with no AKI. However, there was no incremental relationship of age between mild AKI and severe AKI. Significantly fewer patients with severe AKI had history of fever. With increasing severity of AKI, there was a correspondingly graded increase of vomiting, paleness, icterus, and hepatomegaly. Previous history of malaria was not associated with AKI. There was a graded increase in heart rate and decrease in diastolic blood pressure across the severity of AKI. The mean differences in respiratory rate, systolic blood pressure, and axillary temperature were not significant across severity of AKI.

**Table 1:** Demographic and clinical characteristics of the patients stratified by AKI categories.^a^

	No AKI	Mild AKI	Severe AKI	
Characteristics	N = 677	N = 101	N = 46	P value*
**Demographic**				
** Age, years**	33.3 (±13.2)	43.2 (±15.7)	38.9 (±12.6)	**<0.001**
** Gender, Male**	551 (81.4)	89 (88.1)	40 (87)	0.18
**History**				
** History of fever**	672 (99.4)	101 (100)	40 (88.9)	**<0.001**
** Diarrhea**	39 (5.8)	4 (4)	5 (10.9)	0.25
** Vomiting**	229 (33.9)	38 (37.6)	25 (54.3)	**0.017**
**Physical examination**				
** Heart rate, beats/min**	88.5 (±14.1)	92.2 (±17.0)	92.7 (±15.2)	**0.015**
** Respiratory rate, breaths/min**	22.0 (±5.9)	23.4 (±8.3)	23 (±7.6)	0.09
** Systolic blood pressure, mm of Hg**	117.3 (±13.3)	114.4 (±17.4)	116.0 (±17)	0.16
** Diastolic blood pressure, mm of Hg**	75.7 (±9.1)	72.8 (±11.4)	71.9 (±14.4)	**0.001**
** Axillary temperature, °F** b	100.2 (±5.2)	100.3 (±1.7)	99.5 (±1.0)	0.61
** Pallor**	119 (17.6)	21 (20.8)	20 (43.5)	**<0.001**
** Icterus**	154 (22.8)	43 (42.6)	30 (65.2)	**<0.001**
** Splenomegaly**	257 (38.3)	34 (33.7)	30 (66.7)	**<0.001**
** Hepatomegaly**	188 (28.2)	38 (37.6)	34 (75.6)	**<0.001**

a Continuous variables are described as Mean (± Standard Deviation) and categorical variables as n(%).

b To convert temperature to °C = [°F–32]*5/9.

*P values <0.05 are shown in bold.

There was a significant graded decrease in hemoglobin, platelet count, and serum sodium, while in contrast, there was a graded increase in white blood cell (WBC) count, erythrocyte sedimentation rate (ESR), total and direct bilirubin, aspartate aminotransferase (AST), alanine aminotransferase (ALT), alkaline phosphatase (ALP) and serum potassium levels with worsening severity of AKI. While significantly higher proportions of patients with mild and severe AKI had microscopic hematuria (P = 0.007), levels of urinary protein between these groups were not statistically different (P = 0.113). While *P. vivax* and *P. falciparum* were represented equally in the mild AKI category, *P. falciparum* accounted for 80% of the severe AKI category, which was highly significant (Table 2). The parasite index in the available patients showed an increase in the mild and severe AKI categories compared to the no AKI category.

**Table 2:** Laboratory characteristics of the patients stratified by AKI categories.^a^

Characteristics	No AKI	Mild AKI	Severe AKI	P value*
	N = 677	N = 101	N = 46	
**Hematological parameters**				
** Hemoglobin, g/dL**	13.00 [11.1, 14.4]	13.00 [10, 14.6]	10.50 [8.6, 12.6]	**<0.001**
** WBC count, cells/µL**	5200 [4200, 6700]	6100 [5100, 8200]	8500 [5400, 11650]	**<0.001**
** Platelet count, cells/µL**	86000 [51000, 125000]	59000 [30000, 102000]	31000 [20000, 55500]	**<0.001**
** ESR, mm in 1 hour**	28 [14, 51]	35 [16, 82]	68 [33, 107]	**<0.001**
**Plasma glucose, mg/dL**	113 [97, 135]	121 [100, 160]	113 [75, 161]	**0.047**
**Liver function tests**				
** Total bilirubin, mg/dL**	1.5 [1.0, 2.6]	2.4 [1.3, 5.0]	15.6 [5.7, 25.9]	**<0.001**
** Direct bilirubin, mg/dL**	0.5 [0.3, 1.0]	0.9 [0.5, 3.1]	11.8[4.5, 20.9]	**<0.001**
** AST, U/L**	38 [27, 58]	47 [33, 73]	93 [56, 148]	**<0.001**
** ALT, U/L**	37 [24, 57]	45 [29, 63]	51 [27, 70]	**0.021**
** ALP, U/L**	88 [67, 115]	98 [75, 145]	148 [97, 200]	**<0.001**
**Renal function tests and electrolytes**				
** Blood urea, mg/dL**	28 [23, 37]	52 [36, 71]	176 [117, 229]	**<0.001**
** Serum creatinine, mg/dL**	1.1 [1, 1.3]	1.8[1.6, 2.2]	5.2 [4.2, 7.8]	**<0.001**
** Serum sodium, mEq/L**	135 [131, 137]	133 [129, 136]	131 [128, 133]	**<0.001**
** Serum potassium, mEq/L**	3.9 [3.6, 4.3]	4 [3.5, 4.6]	4.6 [4, 5.4]	**<0.001**
**Urine examination**				
** Dipstick protein ≥1+ (30 mg/dL)**	204 (59%)	45 (69.2%)	22 (73.3%)	0.113
** Urine RBC ≥0–10/hpf**	27 (8%)	10 (15.4%)	7 (24.1%)	**0.007**
**Parasite related**				
** Parasite Index, %** b	0.3 [0.2, 0.8]	0.65 [0.2, 2.0]	0.6 [0.3, 2.2]	**0.015**
** Species of Plasmodium**				
*** P. vivax***	450 (66.5%)	50 (49%)	6 (13%)	**<0.001** c
*** P. falciparum***	214 (31.6%)	49 (48.5%)	37 (80.4%)	
** Both**	13 (1.9%)	2 (2%)	3 (6.5%)	

Abbreviations: WBC, White blood cell; ESR, erythrocyte sedimentation rate; AST, aspartate aminotransferase; ALT, alanine aminotransferase; ALP, alkaline phosphatase; RBC, red blood cell; hpf, high power field.

a All continuous variables are described as Median [Inter Quartile Range] and categorical variables as n(%).

b Parasite index was available in 122, 26 and 22 cases with no AKI, mild AKI and severe AKI respectively.

c P value is for Chi square test comparing *P.falciparum* with *P.vivax*.

*P values <0.05 are shown in bold.

Among total 58.59% (498/850) study cohort, glucose –6– phosphate dehydrogenase (G6PD) was estimated to be 13.31±4.06 U/gm Hb, 12.81±3.41 U/gm Hb and 12.94±3.22 U/gm Hb in no AKI, mild AKI and severe AKI categories respectively. Between AKI categories no significant difference in mean G6PD value was determined by one – way ANOVA [F (2, 495) = 0.45, P = 0.64]. A total of 6.02% (30/498) patients were found to have G6PD deficiency in our study cohort. Out of this, 90% [27/30; mean ± SD = 6.9±1.08 U/gm Hb] were in no AKI category, 6.67% [2/30, 7.6 & 7.8 U/gm Hb] in mild AKI category and 3.33% [1/30, 7.8 U/gm Hb] were in severe AKI category. All these patients were treated with weekly dose of primaquine to prevent the occurrence of primaquine induced haemolysis.

#### Progression of renal failure

At least two values of serum creatinine were available in 309 patients (among 200 patients with no AKI, 74 with mild AKI, and 35 with severe AKI on the day of admission). Sixteen patients progressed from no AKI at admission to mild AKI during hospitalization (16/200, 8%). Eight patients (8/74, 10.81%) progressed from mild AKI to severe AKI, and two of them required dialysis. No patients progressed from the no AKI category to severe AKI.

### Demographic, Clinical, and Laboratory Characteristics across AKI Categories among *P. vivax* and *P. falciparum* Cohort (Tables 3 and 4)

*P. vivax* cohort comprised of 450 patients with no AKI, 50 patients with mild AKI and, 6 patients with severe AKI. *P. falciparum* cohort comprised of 214 patients with no AKI, 49 patients with mild AKI and 37 patients with severe AKI. The demographic and clinical characteristics of both cohorts are depicted in Table 3 after excluding mixed infection cases due to relatively very small cohort (n = 18). Male preponderance (≥80%) was noted in both species across all AKI categories. Among both species, patients with AKI were noted to be older than those with no AKI without having any incremental relationship of age difference between the mild and severe AKI categories. History of fever was found to be the same (∼100%) in all AKI categories of *P. vivax* cohort. However, in the *P. falciparum* cohort, significantly fewer patients with severe AKI had history of fever (P<0.001). In the *P. falciparum* cohort, there was a significant graded increase in pallor (P = 0.004), icterus (P<0.001), and hepatomegaly (P<0.001) with increasing severity of AKI, while in the *P. vivax* cohort, there was a significant graded increase in icterus (P = 0.03) and splenomegaly (P = 0.01) with increasing severity of AKI. In addition, the *P. vivax* cohort exhibited a significant graded increase in heart rate (P<0.001) and significant decrease in both systolic blood pressure (P = 0.001) and diastolic blood pressure (P<0.001) across all AKI severity categories. In the *P. falciparum* cohort, none of the vital physical parameters were significantly different across all AKI categories.

**Table 3:** Malaria species wise demographic and clinical characteristics of the patients stratified by AKI categories.^a^

	*Plasmodium vivax* (N = 506)				*Plasmodium falciparum* (N = 300)			
	No AKI	Mild AKI	Severe AKI		No AKI	Mild AKI	Severe AKI	
Characteristics	N = 450	N = 50	N = 06	P value*	N = 214	N = 49	N = 37	P value*
**Demographic**								
** Age, years**	32.3(±13.0)	45.9(±16.2)	40.7(±18.3)	**<0.001**	35.6±13.3	40.2±14.9	38.0±12.0	0.08
** Gender, male**	367(81.6)	48 (96.0)	06 (100.0)	**0.01**	172 (80.4)	39 (79.6)	31 (83.8)	0.89
**History**								
** History of fever**	446 (99.3)	50 (100.0)	06 (100.0)	1.00	213 (99.5)	49 (100.0)	31 (86.1)	**<0.001**
** Diarrhea**	22 (4.9)	02 (4.0)	0	1.00	16 (7.5)	02 (4.1)	05 (13.5)	0.27
** Vomiting**	131 (29.2)	14 (28.0)	04 (66.7)	0.15	93 (43.5)	23 (46.9)	20 (54.1)	0.48
**Physical examination**								
** Heart rate, beats/min**	88.4±13.0	93.5±16.8	107.0±18.6	**<0.001**	88.4±16.3	91.1±17.5	89.4±13.2	0.57
** Respiratory rate, breaths/min**	21.6±4.7	23.3±5.9	22.3±3.4	**0.049**	22.8±7.7	22.4±8.4	22.4±7.3	0.93
** Systolic blood pressure, mm of Hg**	117.7±12.7	112.7±19.7	101.7±17.2	**0.001**	116.2±14.6	116.0±14.8	118.1±16.6	0.76
** Diastolic blood pressure, mm of Hg**	75.9±9.1	73.1±12.7	59.7±21.7	**<0.001**	75.3±9.1	72.7±10.3	73.5±12.5	0.19
** Axillary temperature, °F** b	100.4±5.1	100.3±1.7	100.2±1.0	0.99	99.9±5.6	100.3±1.7	99.4±1.0	0.73
** Pallor**	65 (14.5)	04 (8.0)	01 (16.7)	0.42	50 (23.5)	17 (34.7)	18 (48.6)	**0.004**
** Icterus**	68 (15.1)	14 (28.0)	02 (33.3)	**0.03**	80 (37.6)	28 (57.1)	26 (70.3)	**<0.001**
** Splenomegaly**	161 (35.9)	12 (24.0)	05 (83.3)	**0.01**	91 (43.3)	20 (40.8)	22 (61.1)	0.12
** Hepatomegaly**	104 (23.2)	12 (24.0)	04 (66.7)	0.06	81 (39.1)	24 (49.0)	27 (75.0)	**<0.001**

a Continuous variables are described as Mean (± Standard Deviation) and categorical variables as n(%).

b To convert temperature to °C = [°F–32]*5/9.

*P values <0.05 are shown in bold.

In the *P. vivax* cohort, there was a significant graded decrease in platelet count (P<0.001) and significant increase in WBC count (P = 0.008), plasma glucose (P = 0.005), total and direct bilirubin (P<0.001), AST (P = 0.002), and ALP (P = 0.003) with increasing severity of AKI. In *P. falciparum,* there was a significant graded decrease in hemoglobin (P<0.001), platelet count (P = 0.002) and serum sodium (P = 0.008) with worsening severity of AKI, whereas, a significant graded increase was noted for ESR (P<0.001), total and direct bilirubin (P<0.001), AST (P<0.001), ALP (P = 0.01) and proportion of microscopic hematuria (P = 0.008). As evident in Table 4, in *P. falciparum* cohort a significant difference in mean plasma glucose level was determined by one – way ANOVA [F (2, 262) = 3.256, P = 0.04]. A Tukey post-hoc test revealed that there was a significant (P = 0.047) increase in mean glucose level in mild AKI cohort compared with no AKI cohort.

**Table 4:** Malaria species wise Laboratory characteristics of the patients stratified by AKI categories.^a^

	*Plasmodium vivax* (N = 506)				*Plasmodium falciparum* (N = 300)			
	No AKI	Mild AKI	Severe AKI		No AKI	Mild AKI	Severe AKI	
Characteristics	N = 450	N = 50	N = 06	P value*	N = 214	N = 49	N = 37	P value*
**Hematological parameters**								
** Hemoglobin, g/dL**	13.3 [11.6, 14.6]	13.5 [11.6, 14.6]	12.6 [10.8, 14.4]	0.8	12 [10.1, 13.5]	11.4 [7.6, 14.4]	10.3 [8, 11.7]	**<0.001**
** WBC count, cells/µL**	5200 [4200, 6500]	6100 [5000, 8000]	7000 [3700, 8950]	**0.008**	5300 [4300, 7275]	6100 [5200, 8800]	8900 [5750, 12050]	0.13
** Platelet count, cells/µL**	87000 [56000, 127000]	58500 [23250, 102000]	22000 [18250, 50500]	**<0.001**	81500 [42500, 123750]	63000 [30500, 110500]	34000 [21500, 70000]	**0.002**
** ESR, mm in 1 hour**	22 [12.5, 45]	25 [14, 50]	40 [10, 87]	0.6	33 [18, 63.5]	57 [17, 106]	82.5 [32.7, 120]	**<0.001**
**Plasma glucose, mg/dL**	114 [97.2, 137]	121.5 [105, 153.5]	187 [87, 308]	**0.005**	111 [95, 132]	121 [96, 162]	105 [71, 142]	**0.04**
**Liver function tests**								
** Total bilirubin, mg/dL**	1.4 [0.9, 2.1]	2.0 [1.2, 3.4]	7.9 [2.1, 20.2]	**<0.001**	1.8 [1.1, 3.7]	2.8 [1.2, 6.6]	17.4 [5.6, 29]	**<0.001**
** Direct bilirubin, mg/dL**	0.5 [0.3, 0.7]	0.7 [0.5, 1.7]	6.4 [1.3, 18.2]	**<0.001**	0.6 [0.4, 2.1]	1.5 [0.6, 5.6]	14.1 [4.3, 23.8]	**<0.001**
** AST, U/L**	35 [26, 49.7]	46 [32.7, 65.5]	64 [27.5, 150.5]	**0.002**	48 [29, 84]	50 [32.7, 80]	94 [59, 138]	**<0.001**
** ALT, U/L**	33.5 [23, 51.2]	46 [28, 66.5]	39 [12.5, 70.2]	0.63	45 [29, 75]	42 [30, 56]	45 [30, 67]	0.54
** ALP, U/L**	82 [64, 107]	110.5 [79.5, 155.7]	123 [103.7, 240.7]	**0.003**	96.5 [75, 142.7]	95 [74, 119]	148 [89, 182]	**0.01**
**Renal function tests and electrolytes**								
** Blood urea, mg/dL**	27 [21, 35]	51 [37, 66]	96 [63.2, 190.2]	**<0.001**	30 [25, 45]	63 [34.2, 91.2]	178 [129, 244.5]	**<0.001**
** Serum creatinine, mg/dL**	1.1 [1.1, 1.3]	1.8 [1.6, 2.0]	4.0 [3.3, 9.5]	**<0.001**	1.1 [1.0, 1.3]	1.8 [1.6, 2.3]	5.2 [4.3, 7.4]	**<0.001**
** Serum sodium, mEq/L**	135 [132, 137]	134.5 [130.2, 136]	139 [131, 144]	0.43	134 [130, 137]	132 [128, 135]	131 [128, 132.5]	**0.008**
** Serum potassium, mEq/L**	3.8 [3.5, 4.2]	3.9 [3.6, 4.4]	4.2 [3.8, 4.7]	0.7	4.1 [3.6, 4.4]	4.1 [3.5, 4.7]	4.8 [4.1, 5.4]	0.35
**Urine examination**								
** Dipstick protein ≥1+ (30 mg/dL)**	118 (54.1)	23 (71.9)	03 (60)	0.17	83 (67.5)	22 (66.7)	16 (72.7)	0.91
** Urine RBC ≥0–10/hpf**	18 (8.5%)	05 (15.6%)	0	0.35	08 (6.6%)	05 (15.6%)	06 (28.6%)	**0.008**
**Parasite related**								
** Parasite Index, %** b	0.2 [0.2, 0.6]	0.3 [0.1, 1.3]	–	0.51	0.3 [0.2, 1.1]	1.3 [0.4, 3.5]	0.4 [0.2, 2]	0.06

Abbreviations: WBC, White blood cell; ESR, erythrocyte sedimentation rate; AST, aspartate aminotransferase; ALT, alanine aminotransferase; ALP, alkaline phosphatase; RBC, red blood cell; hpf, high power field.

a All continuous variables are described as Median [Inter Quartile Range] and categorical variables as n(%).

b Parasite index was available in 51, 09 and 00 cases of *P. vivax* with no AKI, mild AKI and severe AKI respectively; 69, 16 and 19 cases of *P. falciparum* with no AKI, mild AKI and severe AKI respectively.

*P values <0.05 are shown in bold.

### Factors Associated with AKI (Tables 5 and 6)

We created two separate regression analyses to identify variables that are independently associated with AKI. In the first analysis, we included variables from patient histories and physical examinations (before any laboratory tests were performed) to identify signs and symptoms that would be harbingers of AKI. In the second analysis, we wanted to associate other laboratory abnormalities that would be present with AKI. On univariate analysis, increasing age, absence of fever, history of vomiting, higher heart rate, lower diastolic blood pressure, and higher respiratory rate, presence of pallor, icterus, and hepatomegaly were associated with AKI. However, with multivariate analysis of factors from history and physical examination, only age, absence of fever, higher heart rate, lower diastolic blood pressure, icterus, and hepatomegaly were independently associated with AKI (Table 5). Icterus was the strongest associated factor for AKI, followed by hepatomegaly and increasing age. Laboratory parameters like lower hemoglobin, higher WBC count, lower platelets, elevated ESR, total and direct bilirubin, AST, ALP, lower serum sodium, higher serum potassium, and infection with *P. falciparum* were significant on univariate analysis. But multivariate analysis of laboratory factors demonstrated that only direct bilirubin was independently associated with AKI (Table 6).

**Table 5:** Univariate and Multivariate Analysis for History and Physical Examination Factors Associated with AKI.

Characteristic	OR (95% CI)*	Adjusted OR (95% CI)*
**Age**	**1.04 (1.03. 1.06)**	**1.05 (1.03, 1.06)**
**Fever**	**0.17 (0.05, 0.63)**	**0.2 (0.05, 0.8)**
**Vomiting**	**1.46 (1.02, 2.11)**	1.13 (0.75, 1.71)
**Diarrhoea**	1.07 (0.50, 2.25)	a
**Heart rate**	**1.02 (1.01, 1.03)**	**1.02 (1.003, 1.030)**
**Respiratory rate**	**1.03 (1.00, 1.05)**	1.03 (0.997, 1.06)
**Systolic blood pressure**	0.99 (0.98, 1.01)	b
**Diastolic blood pressure**	**0.97 (0.95, 0.99)**	**0.97 (0.95, 0.99)**
**Axillary temperature**	0.99 (0.96, 1.03)	a
**Pallor**	**1.81 (1.2, 2.73)**	1.04 (0.64, 1.68)
**Icterus**	**3.34 (2.31, 4.83)**	**2.45 (1.58, 3.82)**
**Splenomegaly**	1.26 (0.88, 1.81)	a
**Hepatomegaly**	**2.48 (1.72, 3.57)**	**1.62 (1.06, 2.46)**
**Spontaneous bleeding**	2.95 (0.95, 9.13)	a

Abbreviations: OR, odds ratio; CI, confidence interval.

a All insignificant variables by univariate model were not included in the multivariate model.

b Systolic blood pressure had multicollinearity with diastolic blood pressure.

* Confidence intervals that do not overlap the null value of OR = 1 are shown in bold font.

**Table 6:** Univariate and Multivariate Analysis for Laboratory Factors Associated with AKI.

Characteristics	OR (95%CI)*	Adjusted OR (95% CI)*
**Hemoglobin**	**0.87 (0.81, 0.93)**	0.92 (0.84, 1.00)
**WBC count/1000**	**1.09 (1.03, 1.16))**	1.01 (0.97, 1.04)
**Platelet count/10000**	**0.92 (0.88, 0.95)**	0.97 (0.93, 1.01)
**Erythrocyte sedimentation rate**	**1.01 (1.01. 1.02)**	a
**Total bilirubin**	**1.17 (1.12, 1.21)**	a
**Direct bilirubin**	**1.21 (1.15, 1.26)**	**1.14 (1.07, 1.20)**
**Aspartate aminotransferase**	**1.01 (1.01, 1.01)**	a
**Alanine aminotransferase**	1.00 (0.999, 1.00)	a
**Alkaline phosphatase**	**1.01 (1.00, 1.01)**	1.00 (0.996, 1.00)
**Serum sodium**	**0.96 (0.92, 0.99)**	0.96 (0.92, 1.00)
**Serum potassium**	1.08 (0.99, 1.17)	0.99 (0.88, 1.12)
***P. falciparum*** ** malaria** b	**3.23 (2.22, 4.69)**	1.55 (0.94, 2.55)

Abbreviations: OR, odds ratio; CI, confidence interval.

a Erythrocyte sedimentation rate was not included because of multicollinearity with hemoglobin; total bilirubin and aspartate aminotransferase were not included because of multicollinearity with direct bilirubin; alanine aminotransferase was not included due to insignificant OR (95% CI) by univariate analysis.

b Comparison with *P.vivax.*

* Confidence intervals that do not overlap the null value of OR = 1 are shown in bold.

### Factors Associated with AKI by Malaria Species (Tables 7 and 8)

As described above for Table 5 and 6, we created two separate regression analyses each for *P. vivax* and *P. falciparum* to identify variables those are independently associated with AKI. On univariate analysis in the *P. vivax* cohort, age, heart rate, respiratory rate, systolic and diastolic blood pressure, and icterus were associated with AKI; however, on multivariate analysis of factors from history and physical examination for the *P. vivax* cohort (Table 7), only age, heart rate, respiratory rate and diastolic blood pressure were independently associated with AKI. Univariate analysis of the *P. falciparum* cohort revealed that age, pallor, icterus and hepatomegaly were associated with AKI, while in multivariate analysis, only absence of fever, icterus and hepatomegaly were independently associated with AKI. Among laboratory parameters, univariate analysis showed higher WBC count, lower platelets, raised total and direct bilirubin, and elevated AST as significant factors for AKI in the *P. vivax* cohort. Furthermore, multivariate analysis (Table 8) showed independent association of higher WBC count and decreased platelets with AKI in the *P. vivax* cohort. Univariate analysis showed decreased hemoglobin, raised ESR, raised total and direct bilirubin, elevated AST, and decreased serum sodium being significant in *P. falciparum* cohort; multivariate analysis showed independent association of decreased hemoglobin and elevated direct bilirubin with AKI in the *P. falciparum* cohort.

**Table 7:** Univariate and Multivariate Analysis for History and Physical Examination Factors Associated with AKI by Malaria Species.

	*Plasmodium vivax*		*Plasmodium falciparum*	
Characteristics	OR (95% CI)*	Adjusted OR (95% CI)*	OR (95% CI)*	Adjusted OR (95% CI)*
**Age**	**1.06 (1.04, 1.08)**	**1.06 (1.04, 1.08)**	**1.02 (1.00, 1.04)**	1.02 (1.00, 1.04)
**Fever**	**–** a	**–** a	**0.07 (0.01, 0.65)**	**0.07 (0.01, 0.69)**
**Diarrhoea**	0.72 (0.16, 3.14)	**–** a	1.09 (0.43, 2.77)	**–** a
**Vomiting**	1.15 (0.63, 2.08)	**–** a	1.30 (0.79, 2.15)	**–** a
**Heart rate**	**1.03 (1.01, 1.05)**	**1.03 (1.01, 1.05)**	1.01 (0.99, 1.02)	**–** a
**Respiratory rate**	**1.07 (1.01, 1.13)**	**1.08 (1.02, 1.15)**	0.99 (0.96, 1.03)	**–** a
**Systolic blood pressure**	**0.97 (0.95, 0.99)**	b	1.00 (0.99, 1.02)	**–** a
**Diastolic blood pressure**	**0.96 (0.94, 0.98)**	**0.95 (0.92, 0.98)**	0.98 (0.95, 1.00)	0.98 (0.95, 1.01)
**Axillary temperature**	0.99 (0.95, 1.05)	**–** a	0.99 (0.95, 1.05)	**–** a
**Pallor**	0.58 (0.22, 1.51)	**–** a	**2.24 (1.31, 3.82)**	1.77 (0.97, 3.19)
**Icterus**	**2.24 (1.19, 4.23)**	1.99 (0.98, 4.05)	**2.81 (1.67, 4.71)**	**1.94 (1.07, 3.49)**
**Splenomegaly**	0.78 (0.43, 1.42)	**–** a	1.28 (0.77, 2.12)	**–** a
**Hepatomegaly**	1.32 (0.71, 2.46)	**–** a	**2.33 (1.39, 3.91)**	**1.92 (1.08, 3.42)**

Abbreviations: OR, odds ratio; CI, confidence interval.

a Univariate analysis did not yield significant difference.

b Variable not included was systolic blood pressure because of multicollinearity with diastolic blood pressure in *P. vivax*.

* Confidence intervals that do not overlap the null value of OR = 1 are shown in bold.

**Table 8:** Univariate and Multivariate Analysis for Laboratory Factors Associated with AKI by Malaria Species.

	*Plasmodium vivax*		*Plasmodium falciparum*	
Characteristics	OR (95% CI)*	Adjusted OR (95% CI)*	OR (95% CI)*	Adjusted OR (95% CI)*
**Hemoglobin**	0.99 (0.88, 1.13)	**–** a	**0.86 (0.78, 0.94)**	**0.86 (0.77, 0.97)**
**WBC count/1000**	**1.17 (1.05, 1.30)**	**1.21 (1.06, 1.38)**	1.03 (0.98, 1.07)	**–** a
**Platelet count/10000**	**0.88 (0.82, 0.94)**	**0.88 (0.81, 0.96)**	**0.95 (0.91, 0.99)**	0.99 (0.94, 1.04)
**Erythrocyte sedimentation rate**	**–** a	**–** a	**1.02 (1.01, 1.02)**	b
**Glucose**	**1.01 (1.00, 1.01)**	**1.01 (1.00, 1.01)**	**1.01 (1.00, 1.01)**	**1.01 (1.00, 1.01)**
**Total bilirubin**	**1.12 (1.04, 1.20)**	b	**1.14 (1.09, 1.19)**	b
**Direct bilirubin**	**1.14 (1.05, 1.24)**	1.06 (0.96, 1.17)	**1.18 (1.11, 1.25)**	**1.15 (1.07, 1.23)**
**Aspartate aminotransferase**	**1.01 (1.00, 1.01)**	1.01 (0.99, 1.01)	**1.01 (1.00, 1.01)**	0.99 (0.99, 1.01)
**Alanine aminotransferase**	1.00 (0.99, 1.01)	**–** a	1.00 (0.99, 1.00)	**–** a
**Alkaline phosphatase**	1.00 (1.00, 1.01)	**–** a	1.00 (0.99, 1.00)	**–** a
**Serum sodium**	0.99 (0.96, 1.04)	**–** a	**0.93 (0.88, 0.97)**	0.95 (0.89, 1.01)

Abbreviations: OR, odds ratio; CI, confidence interval.

a Univariate analysis did not yield significant difference.

b Parameters not included in multivariate analysis due to multicollinearity: For *P. vivax* – total bilirubin with direct bilirubin; For *P. falciparum* – total bilirubin with direct bilirubin and ESR with hemoglobin.

* Confidence intervals that do not overlap the null value of OR = 1 are shown in bold.

### Associated Complications with AKI (Tables 9 and 10)

**Figure 1:**
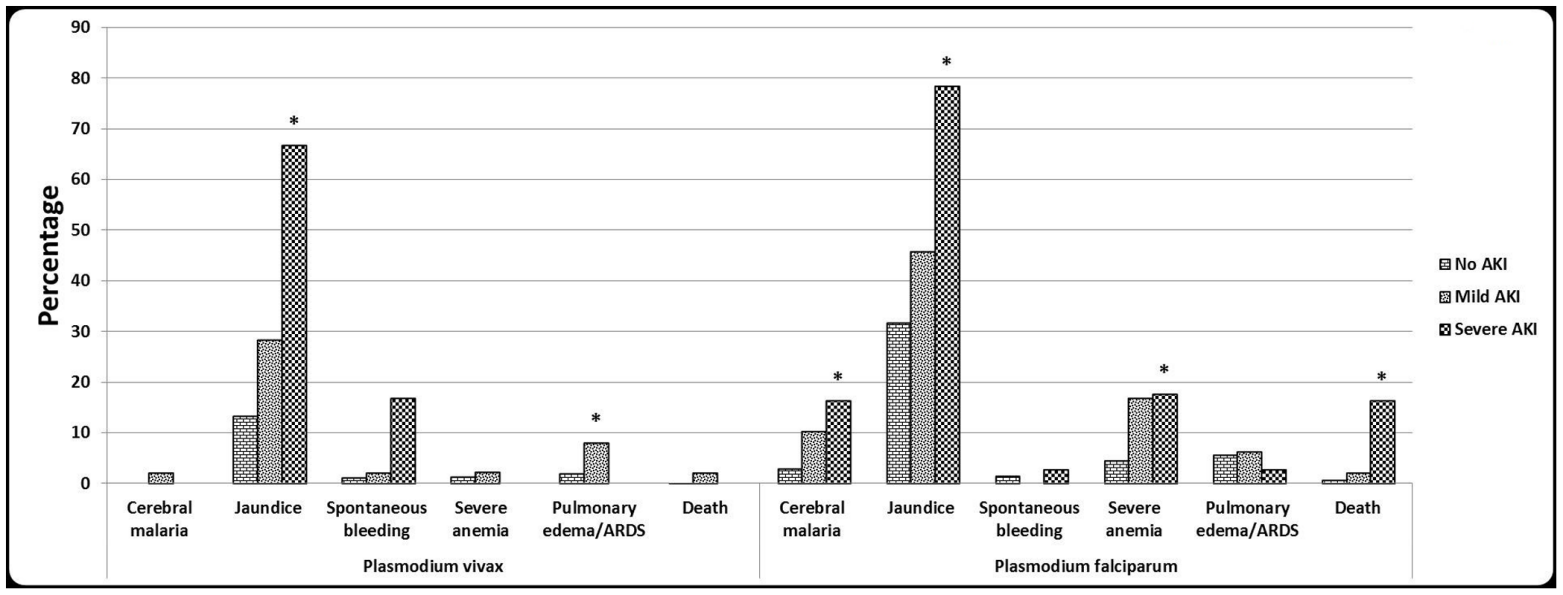
Association of AKI with complications by malaria species. * = P<0.05 across AKI categories.

**Figure 2:**
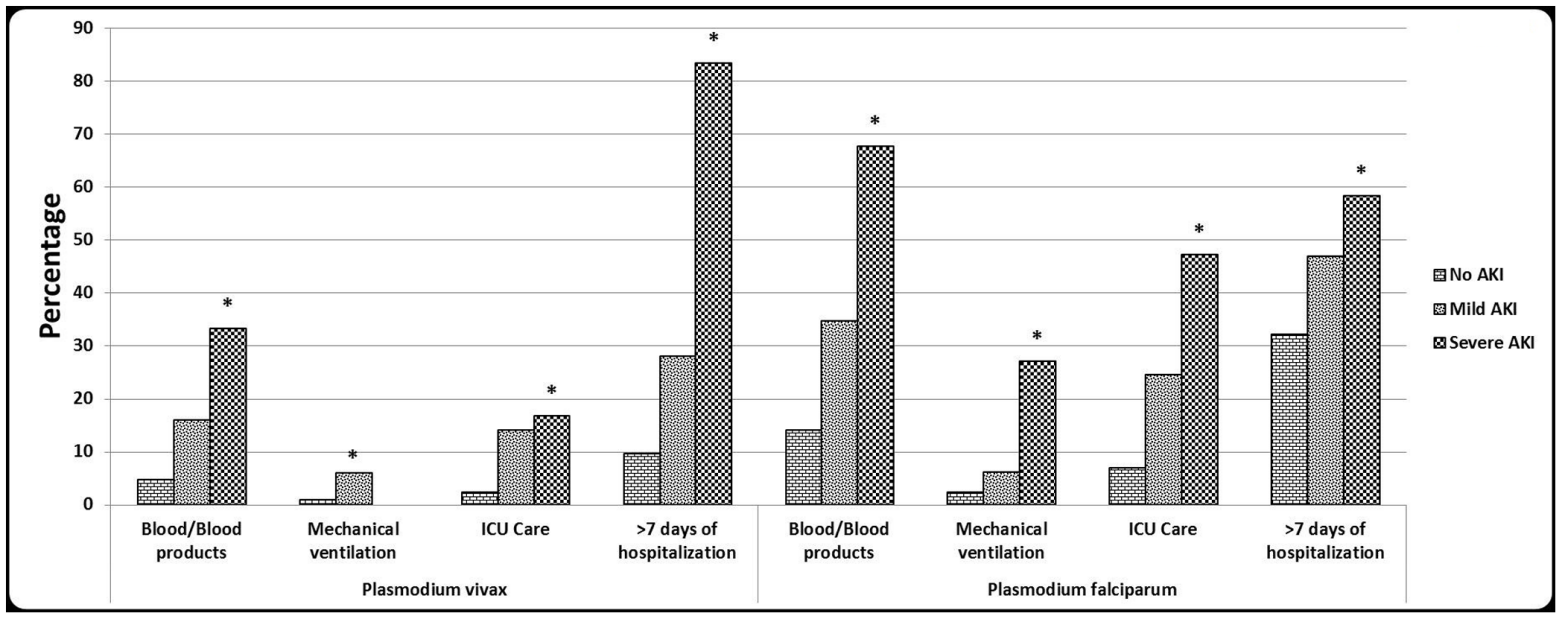
Association of AKI with supportive requirements by malaria species. * = P<0.05 across AKI categories.

Table 9 shows the association of AKI with other complications, wherein complications have been taken as dependent variable and AKI category as independent variable. Patients with severe AKI had higher odds of cerebral malaria, jaundice, severe anemia, and spontaneous bleeding compared to patients with no AKI; even the mild AKI category had significant risk of cerebral malaria, jaundice, severe anemia, and pulmonary edema/ARDS compared to the no AKI category. In total malaria cohort, cerebral malaria, jaundice, spontaneous bleeding, severe anaemia and death were significantly (P<0.04) different among AKI categories. In intra species analysis only jaundice and pulmonary edema/ARDS differed significantly (P<0.05) among AKI categories in *P. vivax*, whereas in *P. falciparum* cohort cerebral malaria, jaundice, severe anaemia and death were significantly different (P<0.05) (Figure 1). Severe AKI as a single organ dysfunction occurred in 8.7% (4/46) of patients and as a part of multi-organ dysfunction in 91.3% (42/46). Even in patients with mild AKI, 51.5% had evidence of other organ dysfunction. Both in total malaria cohort and intra species cohort, patients with mild and severe AKI had incremental requirements for blood products, mechanical ventilation, ICU care and longer hospital stay, except no requirement for mechanical ventilation in severe AKI of *P. vivax* cohort (Figure 2). Hemodialysis was required in 47.8% of cases of severe AKI. No patient with mild AKI had undergone hemodialysis. Patients received a median dialysis of 2.5 (IQR 1.75, 5.52). The severe AKI patients were more likely to die and stay longer in the hospital than those with mild AKI and no AKI. Patients with mild AKI were almost three times as likely to stay longer than 7 days, and those with severe AKI were eight times as likely to stay longer than 7 days compared to patients with no AKI. The median duration of hospitalization were 5 (IQR 4, 7), 7 (IQR 5, 8.5) and 9 (IQR 5.5, 15) days for patients with no, mild, and severe AKI, respectively. The in-hospital mortality was 0.3%, 2%, and 15.2% for patients with no, mild, and severe AKI, respectively. In mild AKI category, pulmonary edema/ARDS, requirement for mechanical ventilation, and death were equally distributed among *P.vivax* and *P. falciparum*, whereas cerebral malaria, jaundice, ICU care, and duration of hospitalization were non-significantly (P>0.05) increased in *P. falciparum* except requirement for blood products (OR (95% CI) = 2.8 (1.1, 7.3) and severe anemia (OR (95% CI) = 9.2 (1.1, 76.8) (Table 10). In the severe AKI category, all complications and supportive requirements as mentioned above were non-significantly (P>0.05) increased in *P. falciparum*, except duration of hospitalization and spontaneous bleeding which were increased in *P. vivax* category.

**Table 9:** Associations of AKI with complications and supportive requirements.

	Mild AKI (N = 101)		Severe AKI (N = 46)	
Characteristics	N (%)	Odds Ratio (95% CI)^a^ *	N (%)	Odds Ratio (95% CI)^a^ *
**Associated complications**				
** Cerebral malaria**	6 (5.9)	**7.1 (2.2, 22.3)**	6 (13.0)	**16.8 (5.2, 54.4)**
** Jaundice**	37 (38.5)	**2.5 (1.6, 4.0)**	36 (78.3)	**14.6 (7.1, 30.2)**
** Spontaneous bleeding**	1(1.0)	0.8 (0.1, 6.8)	4 (8.7)	**7.9 (2.3, 27.5)**
** Severe anemia**	9 (9.3)	**3.9 (1.7, 9.3)**	6 (14.0)	**6.3 (2.3, 17.1)**
** Pulmonary edema/ARDS**	7 (6.9)	**2.4 (1.01, 5.9)**	2 (4.3)	1.5 (0.3, 6.6)
**Supportive requirements**				
** Blood/Blood product**	25(24.8)	**3.9 (2.3, 6.7)**	30 (65.2)	**22.5 (11.5, 43.9)**
** Mechanical Ventilation**	6 (6.0)	**4.7 (1.6, 13.6)**	12 (26.1)	**26.2 (10.3, 66.4)**
** Inotropes**	0	0	2 (4.3)	**6.1 (1.2, 32.4)**
** ICU care**	21 (19.8)	**6.2 (3.3, 11.6)**	21 (46.7)	**21.9 (10.8, 44.3)**
**>7 days of hospitalization**	37 (36.6)	**2.7 (1.7, 4.3)**	28 (62.2)	**7.7 (4.1, 14.6)**
**Death**	2 (2.0)	6.8 (0.9, 48.9)	7 (15.2)	**60.5 (12.2, 300.9)**

Abbreviations: CI, confidence interval; ARDS, acute respiratory distress syndrome; ICU, intensive care unit.

a Reference category was No AKI.

*Confidence intervals that do not overlap the null value of OR = 1 are shown in bold.

**Table 10:** Association of AKI, with complications and supportive requirements by malaria species.

	Mild AKI (N = 99)			Severe AKI (N = 43)		
Characteristics	*P. vivax* N = 50	*P. falciparum* N = 49		*P. vivax* N = 06	*P. falciparum* N = 37	
Associated Complications	N (%)	N (%)	OR (95% CI)#	N (%)	N (%)	OR (95% CI)#
** Cerebral Malaria**	01 (2.0)	05 (10.2)	5.6 (0.6, 49.5)	00	06 (16.2)	–*
** Jaundice**	13 (28.3)	22 (45.8)	2.1 (0.9, 5.1)	04 (66.7)	29 (78.4)	1.8 (0.3, 11.7)
** Spontaneous bleeding**	01 (2.0)	00	–*	01 (16.7)	01 (2.7)	0.1 (0.0, 2.6)
** Severe anemia**	01 (2.1%)	08 (16.7)	**9.2 (1.1, 76.8)**	00	06 (17.6)	–*
** PE/ARDS**	04 (8.0)	03 (6.1)	0.7 (0.1, 3.5)	00	01 (2.7)	–*
**Supportive requirements**						
** Blood/Blood product**	08 (16)	17 (34.7)	**2.8 (1.1, 7.3)**	02 (33.3)	25 (67.6)	4.2 (0.7, 26.0)
** Mechanical Ventilation**	03 (06)	03 (6.2)	1.0 (0.2, 5.4)	00	10 (27)	–*
** Inotropes**	00	00	–*	01 (16.7)	00	–*
** ICU care**	07 (14)	12 (24.5)	1.9 (0.7, 5.6)	01 (16.7)	17 (47.2)	4.5 (0.5, 42.2)
**>7 days of hospitalization**	14 (28)	23 (46.9)	2.3 (0.9, 5.2)	05 (83.3)	21 (58.3)	0.3 (0.0, 2.6)
**Death**	01 (2.0)	01 (2.0)	1.0 (0.1, 16.8)	00	06 (16.2)	–*

Abbreviations: OR, Odds ratio; CI, confidence interval; PE, pulmonary edema; ARDS, acute respiratory distress syndrome; ICU, intensive care unit.

–*Statistics could not be computed.

# Confidence intervals that do not overlap the null value of OR = 1 are shown in bold.

Mortality was seen in total nine patients with AKI. One *P. vivax* patients with mild AKI who died also had PE/ARDS. One *P. falciparum* patient with mild AKI who died also had cerebral malaria, PE/ARDS, severe anaemia and jaundice. Among six *P. falciparum* patients with severe AKI who died, one had PE/ARDS, another had both severe anaemia and jaundice, and other four had jaundice. Single patient with mixed infection and severe AKI who died also had PE/ARDS and jaundice. Out of seven patients with severe AKI (six *P. falciparum* and one mixed infection) haemodialysis was administered to three severe AKI patients.

## Discussion

Most previous publications of AKI in malaria have described the clinical course of severe AKI in the setting of malaria, and several of these only focused on the *P.falciparum* species [6], [10], [12], [13], [14]. Our objective was to determine whether less severe forms of AKI had an impact on morbidity and mortality in patients hospitalized for malaria. Our results illustrate that even patients with mild AKI had higher odds of complications, such as cerebral malaria, jaundice, severe anemia, and pulmonary edema/ARDS, compared to patients who did not develop AKI. Similarly, mild AKI increased the need for supportive requirements and markedly increased the length of hospital stay. However, mild AKI was not associated with higher mortality.

The occurrence of severe AKI in malaria is recognized to impart a grim prognosis [10]. The incidence of severe AKI in the present cohort of acute malaria was 5.58%, and 47.8% of the severe AKI cases required hemodialysis. The low incidence of severe AKI compared to other studies can be explained by our inclusion of the full spectrum of hospitalized malaria patients and the predominance of *P.vivax* species [12]. The requirement of dialysis was similar to that of Thanachartwet *et al.* [12], although lower than the reported 73–80% by other studies [6], [13], [15]. The 15.2% severe AKI mortality rate was in concordance with some studies, [4], [6] but lower than the range of 26–32% reported by others [10], [12]. Severe AKI was associated with higher odds of complications, requirement of supportive care, and duration of hospitalization in conformity with other studies [10], [13]. In view of the high mortality associated with severe AKI and associated morbidity even with mild AKI, it is paramount to detect mild AKI early to prevent progression to severe AKI. Hence, we decided to identify the factors associated with mild and severe AKI.

### Factors Associated with AKI in Malaria

On histories and physical exams, increasing age, heart rate, absence of fever, lower diastolic blood pressure, and presence of icterus and hepatomegaly were independently associated with presence of AKI. This information can be applied in any healthcare setting to identify patients who have a higher likelihood of AKI in the presence of malaria. Our analysis, which included laboratory parameters, confirmed that hepatic dysfunction such as direct bilirubinemia is independently associated with AKI. Thus, independent association of hepatomegaly and bilirubin suggest that hepatic complications are closely tied to development of AKI. With our study design, it is impossible to discern if AKI preceded liver abnormalities or vice versa. Future studies should investigate the temporal association using more sensitive measures such as biomarkers of AKI that are elevated much before serum creatinine [16]. Hyperbilirubinemia plays an important role in the pathogenesis of AKI. It alters the renal haemodynamics [17], [18] and depresses the cardiac function [19]. Bilirubin is known to be toxic for renal tubular cells as shown in obstructive jaundice patients in the presence of additional factors like hypovolemia and hypoxia [20].

Patients with history of fever probably sought medical attention earlier than those without history of fever and thereby were protected against severe AKI. However, we cannot rule out probable recall bias about fever by the patients. We found diastolic blood pressure to be negatively associated with AKI. Lower diastolic blood pressure is a reflection of systemic vasodilatation which results in decreased effective blood volume and reduced blood flow to the kidney [21], [22]. This would suggest that a majority of the early mild AKI is due to pre-renal azotemia, providing an opportunity to intervene early to prevent progression of mild AKI to severe AKI. Heart rate was positively associated with AKI which is a reflection of shock. We also did not find any association with parasite index. Moreover, Nacher *et al.* has demonstrated that schizont count and their relative proportion to ring forms, were not risk factors for severe AKI, implying sequestration was not the most important determinant of severe AKI in malaria [23].

### Factors Associated with AKI by Malaria Species

#### *Plasmodium vivax*

Table 7 shows association of age, heart rate, and respiratory rate with AKI, but negative association with diastolic blood pressure. Thus, it appears that AKI in *P. vivax* is primarily due to haemodynamic alteration in kidney.

#### *Plasmodium falciparum*

Hyperbilirubinemia is associated with AKI in *P. falciparum*. This is supported by association of AKI with icterus, hepatomegaly (Table 7), and direct bilirubin. Negative association of AKI with hemoglobin (Table 8) reflects haemolysis contributing to hyperbilirubinemia.

We would like to emphasize that although there are increasing reports of *P. vivax* associated AKI, we observed that the *P. vivax* contributed to half of the mild AKI category but only to 13% of the severe AKI category [8], [9]. However, because *P. vivax* is the predominant cause of malaria in Southeast Asia, the absolute contribution to severe AKI may still be substantial. Thus, early detection and treatment of mild AKI cases with *P. vivax* may in fact significantly reduce the prevalence of severe AKI in these regions.

### Strengths and Weaknesses of the Study

The present study has significant strengths. It is the first report of AKI stratified by severity in a large cohort of consecutive patients hospitalized with malaria. The severe AKI definition corresponds to WHO criteria for acute renal failure and enables comparison with published literature. This study is the largest cohort of acute malaria patients with a 60% prevalence of *P.vivax* that has provided rigorous analysis of associated factors of AKI. However, the retrospective nature of the study has its inherent limitations. Although we found absence of fever to be associated with AKI, a probable recall bias resulting in this finding could not be ruled out due to lack of data on duration of illness/delay in presentation. We did not have historical or in-hospital urine output data systematically recorded. Hence, the potential of oliguria as a risk factor for AKI could not be assessed. Outpatient baseline serum creatinine values were not available, and at least two values of serum creatinine were available only in about a third of the cohort. Thus, we could not define AKI by contemporary risk, injury, failure, loss, and end stage kidney disease (RIFLE) or acute kidney injury network (AKIN) criteria. With our study design, as the timing of the AKI occurrence or the mechanism of AKI could not be discerned, these should be further evaluated by a prospective design with timely assessment of a panel of most sensitive and specific renal biomarkers. Hemodialysis done on patients was based on clinicians’ judgement in consultation with nephrologists and was not based on pre-defined hard end points. Additionally, we do not have data on haemodialysis initiation timings for any patient. Data regarding metabolic acidosis, hemoglobinuria, reticulocyte count, coagulation parameters, and measure of depth of coma in cerebral malaria-like Glasgow coma scale, and empiric antibiotics used were not available, and parasite density was available only in a few patients. Speciation was done microscopically and not by more sensitive polymerase chain reaction tests.

## Conclusion

Age, absence of fever, lower diastolic blood pressure, heart rate, icterus, hepatomegaly, and direct bilirubin were factors found to be associated with AKI in our study. The results confirm that severity of AKI has prognostic value in morbidity and mortality in acute malaria. Future studies should investigate if even earlier detection with novel biomarkers, before increase in serum creatinine, would alter the epidemiology of this preventable complication. It remains a possibility that early recognition of AKI and prompt, aggressive treatment to avert disease progression may improve the outcomes in the setting of malaria.
